# An executive summary on the Global conceptual definition of Sarcopenia

**DOI:** 10.1007/s40520-024-02798-4

**Published:** 2024-07-27

**Authors:** Ben Kirk, Peggy M. Cawthon, Hidenori Arai, José A. Ávila-Funes, Rocco Barazzoni, Shalender Bhasin, Ellen F. Binder, Olivier Bruyère, Tommy Cederholm, Liang-Kung Chen, Cyrus Cooper, Gustavo Duque, Roger A. Fielding, Jack Guralnik, Douglas P. Kiel, Francesco Landi, Jean-Yves Reginster, Avan A. Sayer, Marjolein Visser, Stephan von Haehling, Jean Woo, Alfonso J. Cruz-Jentoft, The Global Leadership Initiative in Sarcopenia (GLIS) Group

**Affiliations:** 1 Department of Medicine, Western Health, Melbourne Medical School, University of Melbourne, St Albans, Melbourne, VIC Australia; 2 Australian Institute for Musculoskeletal Science (AIMSS), University of Melbourne and Western Health, St Albans, Melbourne, VIC Australia; 3 California Pacific Medical Center, Research Institute, 550 16th Street, Second Floor, San Francisco, CA 94143 USA; 4 Department of Epidemiology and Biostatistics, University of California San Francisco, San Francisco, CA USA; 5 National Center for Geriatrics and Gerontology, Obu, Aichi Japan; 6 Department of Geriatrics, Instituto Nacional de Ciencias Médicas y Nutrición Salvador Zubirán, Mexico, Mexico; 7 Univ. Bordeaux, Inserm, Bordeaux Population Health Research Center, UMR 1219, 33000 Bordeaux, France; 8 Department of Medical, Surgical and Health Sciences, University of Trieste, Trieste, Italy; 9 Boston Claude D. Pepper Older Americans Independence Center, Brigham and Women’s Hospital, Harvard Medical School, Boston, MA USA; 10 Division of General Medicine and Geriatrics, School of Medicine, Washington University in St. Louis, St. Louis, MO USA; 11 WHO Collaborating Center for Public Health Aspects of Musculo-Skeletal Health and Ageing, Division of Public Health, Epidemiology and Health Economics, University of Liège, Liège, Belgium; 12 Department of Sport and Rehabilitation Sciences, University of Liège, Liège, Belgium; 13 Department of Public Health and Caring Sciences, Clinical Nutrition and Metabolism, Uppsala University, Uppsala, Sweden; 14 Theme Inflammation and Ageing, Karolinska University Hospital, Stockholm, Sweden; 15 Center for Geriatrics and Gerontology, Taipei Veterans General Hospital, Taipei, Taiwan; 16 Center for Healthy Longevity and Aging Sciences, National Yang Ming Chiao Tung University, Taipei, Taiwan; 17 MRC Lifecourse Epidemiology Unit, University of Southampton, Southampton, UK; 18 Department of Epidemiology, University of Oxford, Oxford, OX UK; 19 Bone, Muscle and Geroscience Group, Research Institute of the McGill University Health Centre, Montreal, QC Canada; 20 Dr. Joseph Kaufmann Chair in Geriatric Medicine, Department of Medicine, McGill University, Montreal, QC Canada; 21 Nutrition Exercise, Physiology, and Sarcopenia Laboratory, Jean Mayer U.S. Department of Agriculture Human Nutrition Research Center On Aging, Tufts University, Boston, MA USA; 22 Department of Epidemiology and Public Health, University of Maryland School of Medicine, Baltimore, MD USA; 23 Hinda and Arthur Marcus Institute for Aging Research, Hebrew SeniorLife, Department of Medicine Beth Israel Deaconess Medical Center, Harvard Medical School, Boston, MA USA; 24 Fondazione Policlinico Universitario “Agostino Gemelli” IRCCS, 00168 Rome, Italy; 25 WHO Collaborating Center for Epidemiology of Musculoskeletal Health and Aging, Liège, Belgium; 26 Chair for Biomarkers of Chronic Diseases, College of Science, King Saud University, Riyadh, Kingdom of Saudi Arabia; 27 AGE Research Group, NIHR Newcastle Biomedical Research Centre, Newcastle Hospitals and Faculty of Medical Sciences, Newcastle University, Newcastle upon Tyne, UK; 28 Department of Health Sciences, Faculty of Science, Vrije Universiteit Amsterdam, Amsterdam, The Netherlands; 29 The Amsterdam Public Health Research Institute, Amsterdam, The Netherlands; 30 Department of Cardiology and Pneumology, University Medicine Göttingen (UMG), Göttingen, Germany; 31 German Centre for Cardiovascular Research (DZHK), Partner Site Göttingen, Göttingen, Germany; 32 Department of Medicine and Therapeutics, The Chinese University of Hong Kong, Hong Kong, China; 33 Servicio de Geriatría, Hospital Universitario Ramón y Cajal (IRYCIS), Madrid, Spain

**Keywords:** Sarcopenia, Muscle mass, Muscle strength

## Objective

To address a significant gap in research and clinical practice by developing the global conceptual definition of Sarcopenia.

## Methodology

### Study design

Two-phase Delphi study with participants from leading international musculoskeletal organisations and sarcopenia societies [1]. A steering committee was formed of representatives from each continent/region (Asia, Europe, North America, South America, New Zealand/Australia). The steering committee then developed a glossary of research terms on sarcopenia [2]. Following this, a list of statements on critical aspects of sarcopenia was developed and finalised.

### Participants

Invitations to complete an online survey were sent to international experts from academic, industry, and healthcare professions. All participants including steering committee members completed a declaration of interest form prior to participation.

### Statistical analysis

Prespecified criteria were set by the steering committee including a two-phase study design and an acceptable threshold of > 80% for accepted statements [1].

## Findings

### Participants

107 participants (64% men) from 29 countries and across 7 continents/regions completed the Delphi. The majority were residing in Europe (40%), Asia (22%) and North America (19%). Participants reported their primary role as academic professionals (76 [71%]), health professional (23 [22%), industry professionals (3 [2%]), or other-mixed (5 [5%]).

### Accepted and rejected statements

Across the three facets of sarcopenia; ‘general aspects’, ‘components’ and outcomes’, 20 statements were accepted with strong agreement and 4 rejected with low agreement.

**Fig. 1:**
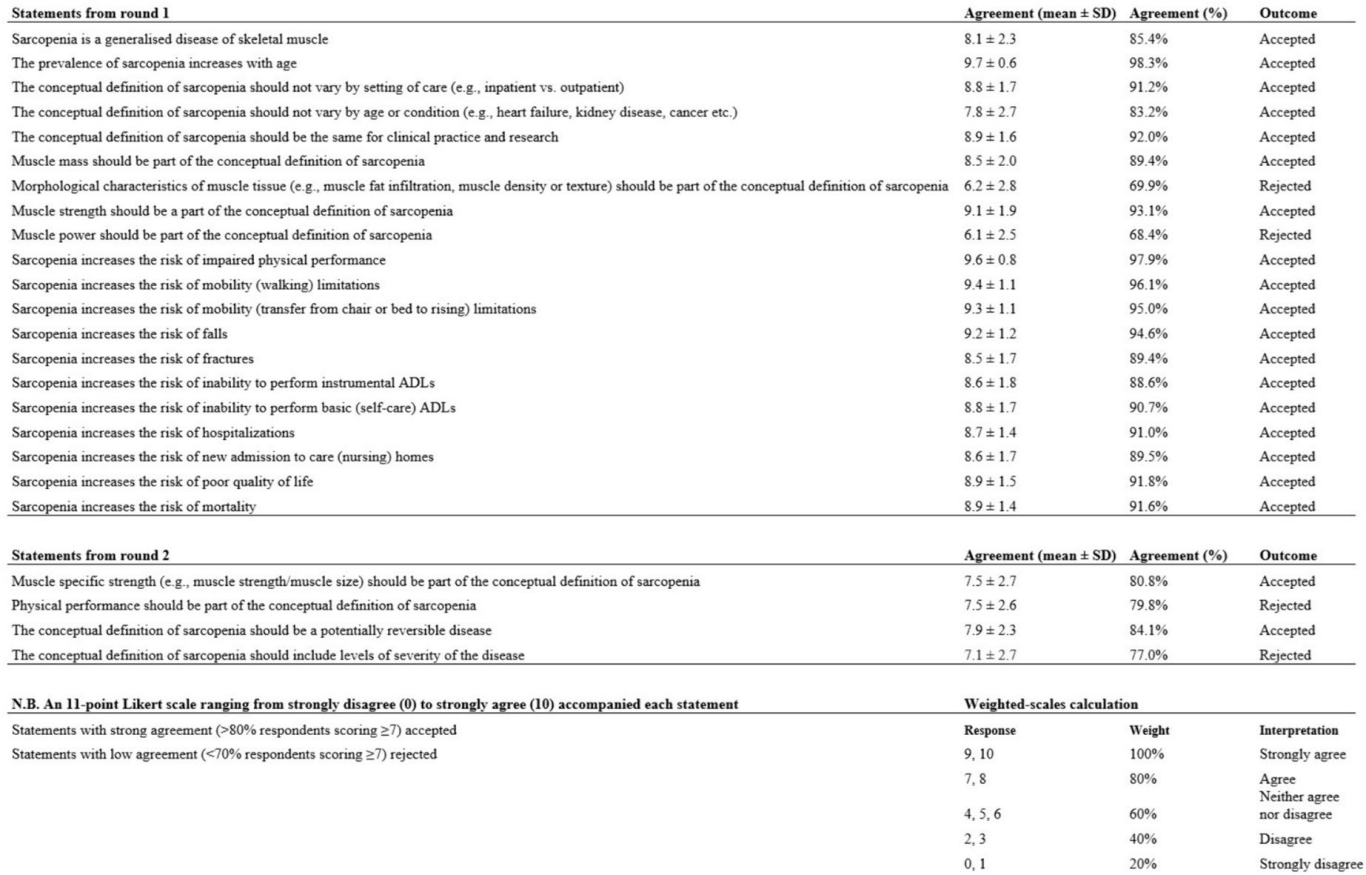
The list of accepted and rejected statements from the Delphi study (*n* = 107 participants). Sourced from Kirk et al. [1]

Figure 1 shows the list of accepted and rejected statements

### Key points

#### General aspects

Sarcopenia is a disease of skeletal muscle; the definition will be the same for research and clinical practice; the definition will not depend on age, setting of care or clinical condition.

#### Components

The definition will comprise of both reduced muscle mass and strength, as well as muscle-specific strength. Physical performance will not be considered as a component of sarcopenia but instead as an outcome.

#### Outcomes

Sarcopenia increases a host of adverse health outcomes (e.g. fragility fractures, disability, poor quality of life), nursing home admissions and premature mortality. Impaired physical performance will be considered a measurable outcome of sarcopenia.

**Fig. 2:**
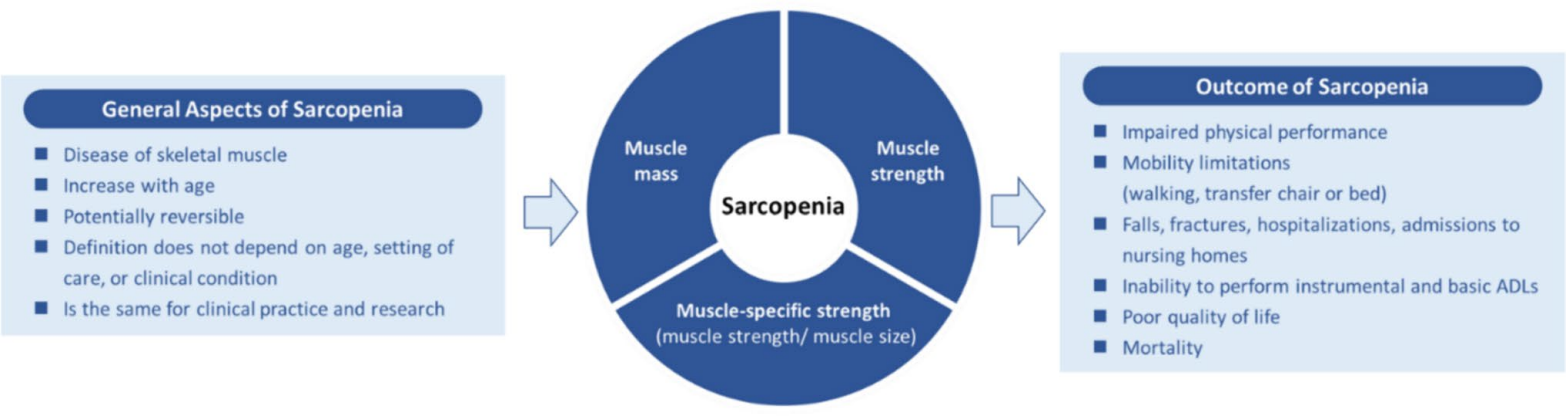
The global conceptual definition of Sarcopenia. Sourced from Kirk et al. [1]

Figure 2 shows the global conceptual definition of sarcopenia that will be used to develop an operationalized definition

## Summary

The global conceptual definition of sarcopenia has been developed using an International Delphi Study including academic, industry and healthcare professionals from leading musculoskeletal organisations and sarcopenia societies. This inclusive approach will serve as a strong backbone to develop an operational definition of sarcopenia for research and clinical settings.

## Future directions

Three working groups are currently underway to develop an operational definition of sarcopenia. These working groups, comprising of experts on specific sarcopenia topics including components and outcomes of the disease, are expected to finalise the operational definition in the coming year(s).

The Global Leadership Initiative in Sarcopenia (GLIS) represents a significant advancement in the field to improve the prevention, diagnosis and treatment of this muscle disease.

## Data Availability

No datasets were generated or analysed during the current study.
